# Ultrafast Study of Interfacial Charge Transfer Mechanism in Assembled Systems of CsPbBr_3_ and Titanium Dioxide: Size Effect of CsPbBr_3_

**DOI:** 10.3390/nano15141065

**Published:** 2025-07-09

**Authors:** Ying Lv, Menghan Duan, Jie An, Yunpeng Wang, Luchao Du

**Affiliations:** 1 Institute of Atomic and Molecular Physics, Jilin University, Changchun 130012, China; yinglv23@mails.jlu.edu.cn (Y.L.); duanmh21@mails.jlu.edu.cn (M.D.); anjie18@mails.jlu.edu.cn (J.A.); 2 Jilin Provincial Key Laboratory of Applied Atomic and Molecular Spectroscopy, Jilin University, Changchun 130012, China; 3 State Key Laboratory of Luminescence and Applications, Changchun Institute of Optics, Fine Mechanics and Physics, Chinese Academy of Sciences, Changchun 130033, China

**Keywords:** perovskite, CsPbBr_3_, transient absorption, ultrafast dynamics, size effect, interfacial carrier transfer

## Abstract

Lead halide perovskite quantum dots, also known as perovskite nanocrystals, are considered one of the most promising photovoltaic materials for solar cells due to their outstanding optoelectronic properties and simple preparation techniques. The key factors restricting the photoelectric conversion efficiency of solar cell systems are the separation and transmission performances of charge carriers. Here, femtosecond time-resolved ultrafast spectroscopy was used to measure the interfacial charge transfer dynamics of different sizes of CsPbBr_3_ assembled with TiO_2_. The effect of perovskite size on the charge transfer is discussed. According to our experimental data analysis, the time constants of the interfacial electron transfer and charge recombination of the assembled systems of CsPbBr_3_ and titanium dioxide become larger when the size of the CsPbBr_3_ nanocrystals increases. We discuss the physical mechanism by which the size of perovskites affects the rate of charge transfer in detail. We expect that our experimental results provide experimental support for the application of novel quantum dots for solar cell materials.

## 1. Introduction

Since the birth of organic–inorganic hybrid perovskite solar cells, the perovskite system has attracted wide attention from all walks of life, but due to the fact that the high polarity of water molecules can destroy the bonds between the A-site and perovskite octahedral, resulting in structural instability, and the existence of oxygen or ultraviolet rays can cause perovskite to undergo further irreversible degradation [1,2,3], many researchers are working to improve both perovskite’s stability conditions and photoelectric performance. All-inorganic lead halide perovskite quantum dots have received great research interest in photovoltaic applications because compared to other solar cells, perovskite quantum dots have higher efficiency and possess various exciting properties, including (i) great defect tolerance [4,5], (ii) a long carrier lifetime [6], (iii) the stabilization of unstable crystallographic phases through surface strain [7], and (iv) superior photoluminescence quantum yields [8,9,10,11,12]. Based on these advantages, the power conversion efficiency of perovskite quantum dots solar cells is higher than that of other perovskite solar cells, and all-inorganic lead halide perovskite quantum dots solar cells have achieved over 15% high efficiency, demonstrating the enormous potential of photovoltaics [13]. Therefore, perovskite quantum dots are very attractive for certain potential applications, such as perovskite quantum dot solar cells, detectors, scintillators, and LEDs.

In order to elucidate the basic mechanism for improving the photoelectric conversion efficiency of solar cells, the transient characteristics and dynamics of photoexcited carriers in all-inorganic perovskites can be studied using ultrafast spectroscopy technology. There are several processes involved in carrier dynamics after photoexcitation, including hot-carrier cooling, carrier diffusion, charge recombination, and other processes [14]. Samanta et al. studied the spectral and temporal characteristics of CsPbBr_3_, CsPbBr_2_I, CsPbBr_1.5_I_1.5_, and CsPbI_3_ using femtosecond transient absorption (fs-TA) techniques. The components of hot-carrier cooling, carrier trapping, and the recombination processes were distinguished, and it was observed that increasing iodide content in perovskites does not affect electron trapping but creates additional hole trapping centers [15]. Tang et al. launched a compositional engineering strategy, using inorganic CsPbBr_3_ perovskite films (CsTM_1-X_Pb_X_Br_3_, TM^2+^ = Mn^2+^, Ni^2+^, Cu^2+^, and Zn^2+^) to reduce the grain boundary and decrease the trap density of states in the perovskite film, which leads to a reduced charge recombination and enhanced charge extraction [16]. Bardhan et al. showed that a higher Cl content in perovskite leads to a lower photoluminescence quantum yield and the more rapid radiative decay of carriers, as well as a reduction in the band gap renormalization energy. They found that the Auger process is more dominant than the trap-assisted recombination process in Cl-replaced mixed-halide NCs [17]. Wang et al. compared the band edge carrier dynamics and diffusion processes of MAPbBr_3_ and CsPbBr_3_ single crystal microplates. They found that the latter has a higher bulk-free carrier recombination rate and diffusion constant, which improves the performance of photoelectric conversion not only in terms of structural stability but also in terms of carrier transmission [18]. Lv et al. studied the carrier dynamics and the diffusion process of LiBr-CsPbBr_3_ thin films by means of transient absorption techniques. Their study found that the trapping probability of hot carriers decreases, thus increasing the carrier diffusion rate. The effect of hot-carrier trapping on carrier diffusion was demonstrated, and new insights into the carrier dynamics of all-inorganic CsPbBr_3_ perovskite films were provided [19]. Several of the research groups mentioned above have studied the transient properties and dynamics of photoexcited carriers in all-inorganic perovskites. However, the detailed mechanisms are still unclear. The focus of our work will be the further investigation of the physical mechanisms. Our group has demonstrated that the electron transfer and charge recombination time constants change with increasing I content by studying the ultrafast spectrum of the influence of halogen ion composition on interfacial charge transfer in assembled systems of CsPbBr_3_ and titanium dioxide [14]. Furthermore, previous research conducted by our group examined the transient dynamic process of assembled systems of CsPbBr_3_ and titanium dioxide with varying Mn^2+^ doping concentrations by using femtosecond transient absorption spectroscopy. It is proposed that with an increase in Mn^2+^ doping, not only can the relaxation orbitals of charge carriers and defect state density be increased, but also the band gap of CsPbBr_3_ can be regulated, and the photoelectric conversion efficiency can be effectively improved [20]. In addition, our group has also investigated the charge carrier dynamics of perovskites doped with Mn^2+^ and undoped perovskites under varying pressure conditions. Our findings indicated that the lattice compression energy of CsPbBr_3_ doped with a manganese ion is greater than that of CsPbBr_3_ alone. Furthermore, the incorporation of manganese ions enhances the stability of the perovskite under pressure [21].

One of the core research directions to promote the development of photovoltaic applications is the size regulation of perovskite. Size control plays a crucial role in perovskite quantum dot solar cells. Du et al. investigated the charge transfer mechanism of gold nanodots assembled with TiO_2_ systems, and they found that the size of TiO_2_ affects the rate of charge recombination but not the electron injection efficiency [22]. Song et al. and Pan et al. discovered that adjusting the size of CsPbX_3_ quantum dots (QDs) could modulate their luminescence wavelength and photoluminescence (PL) yield. However, they do not discuss the photophysical processes associated with defects in size [23,24]. Furthermore, the influence of the nanoscale size of CsPbBr_3_ on photoluminescence has been reported, which helps explain the recombination pathways and dynamics of photogenerated charge carriers [25,26,27,28]. However, there is relatively little research on the effects of CsPbBr_3_ size on ultrafast dynamics such as hot-carrier relaxation, electron transfer, and recombination processes. Therefore, we further investigated the charge transfer mechanism of CsPbBr_3_ at different sizes. By precisely controlling the size of perovskite quantum dots, the photoelectric performance and stability of the battery can be significantly optimized. This endows researchers with the powerful ability to regulate the band gap of materials to maximize the capture of solar light and improves the photoelectric conversion efficiency by affecting carrier behavior, carrier transport, and recombination dynamics. At the same time, controlling the morphology and size of quantum dots provides a unique approach to solving the stability problem of perovskite materials. Therefore, revealing the intrinsic correlation between the size and the interfacial charge transfer dynamics of CsPbBr_3_ provides a new perspective on the photophysical mechanism of perovskite and opens up a more dimensional regulatory pathway for designing highly efficient, stable, and functionalized perovskite quantum dot solar cells.

In this paper, the femtosecond time-resolved ultrafast spectroscopy technology was used to study the complex process of the separation and recombination of photogenerated electrons in all-inorganic CsPbBr_3_ perovskite of different sizes assembled with titanium dioxide systems. The effect of different crystal sizes on photogenerated carrier dynamics is discussed using singular value decomposition (SVD) and global fitting. The transient processes are discussed in detail by separately examining the hot-carrier cooling, electron transfer, and charge recombination processes based on the fitting results. Our experimental results provide physical-theoretical guidance for the design of high-efficiency solar cells by providing insight into carrier–phonon dynamics. We believe that our results can also provide convincing experimental evidence for the optimization and design of novel solar cells.

## 2. Materials and Methods

### 2.1. Preparation of Mesoporous TiO_2_ Thin Films

A certain amount of titanium dioxide (TiO_2_ (anatase) from Aladdin Reagent, Inc. (Pico Rivera, CA, USA)) with a particle size of 40 nm was dissolved in an anhydrous ethanol solution using ultrasonic waves. Then, 20 microliters of the resulting TiO_2_-ethanol mixture was deposited onto a thoroughly cleaned and dried glass substrate and spin-coated at a rotational speed of 1000 rpm for a duration of 30 s. Because the thickness of the film can be controlled by adjusting the spin-coating speed and concentration of the sample [29], we used the same concentrations, spin-coating speeds, and times when preparing the TiO_2_ films. The thicknesses of the TiO_2_ films produced in the experiment were essentially the same. Finally, the mesoporous TiO_2_ film layer was annealed on a digitally regulated heating plate at 450 °C for 1 h.

### 2.2. Preparation of CsPbBr_3_ Perovskite Nanocrystals

The synthesis of CsPbBr_3_ perovskite nanocrystals was accomplished by a high-temperature thermal injection technique, and some slight adjustments were made on the foundation of the previous preparation method [23,30,31]. The first step was to prepare a Cesium-Oleate (Cs-Oleate) precursor solution. Cesium carbonate (0.35 g, Cs_2_CO_3_, Sigma-Aldrich, St. Louis, MO, USA, 99.9%), octadecene (20 mL, ODE, Sigma-Aldrich, 90%), and oleic acid (1.25 mL, OA, Sigma-Aldrich, 90%) were added to a 50 mL three-necked round bottom flask. The temperature of the reaction solution was maintained at 150 °C, and stirring was continued until all Cs-Oleate precursors were dissolved. The resulting transparent solution was preserved. The second step was the preparation of CsPbBr_3_ nanocrystals. Octadecene (12 mL, ODE, Sigma-Aldrich, 90%) was charged into a 50 mL three-necked round-bottomed flask, and the temperature of the solution was controlled at 100 °C. A total of 0.5 mmol of PbBr_2_ (0.1835 g, Sigma-Aldrich, 98%) was added, and the reaction was continuously stirred under the condition of continuous N_2_ inflow for 1 h. Then, the reaction temperature was set at 140 °C, and 1.5 mL of oleic acid and 1.5 mL of oleylamine (OLA, Sigma-Aldrich, 70%) were injected. A total of 1 mL of Cs-Oleate precursor solution was removed and rapidly injected into the reaction mixture when the solution became clear at 140 °C. Given that Cs-Oleate precipitated from ODE at room temperature, it had to be preheated to 100 °C before injection. The reaction was stopped by immersing the flask in an ice-water mixture, and a light green color appeared within 5 s. A crude solution of CsPbBr_3_ nanocrystals was obtained. In order to obtain CsPbBr_3_ nanocrystals of different sizes, we set the temperatures of the preparation process to 150 °C, 160 °C, 170 °C, 180 °C, and 190 °C, respectively.

Then, the crude solution of CsPbBr_3_ nanocrystals prepared at different reaction temperatures was washed twice: In the first wash, the crude solution was taken, methyl acetate (MeOAc) was added at a 1:2 solution volume ratio (crude solution: methyl acetate = 1:2), and the precipitate was dispersed in 3 mL of n-hexane solvent by centrifugation at 4000 rpm for 10 min. In the second wash, methyl acetate (MeOAc) was added to the above solution at a volume ratio of 1:2 and centrifuged at 4000 rpm for 10 min. The precipitate was dispersed in 5 mL of hexane, and then the purified CsPbBr_3_ nanocrystal solution was obtained.

### 2.3. Femtosecond Transient Absorption Spectroscopy

In order to better understand the interfacial charge transfer mechanism of CsPbBr_3_ of different sizes and titanium dioxide, the transient absorption spectra were measured using femtosecond pump–probe experiments. The light source was an amplified mode-locked Ti:sapphire laser (Libra-USP-HE, Coherent Inc., Santa Clara, CA, USA, center wavelength 800 nm, repetition frequency 1 kHz). The 800 nm fundamental beam from the Ti:sapphire amplifier was frequency-doubled to 400 nm through a β-barium borate (BBO) crystal to generate the pump pulse. The probe beam was passed through an optical delay and focused on a sapphire crystal to produce a white light continuum. At the same time, to obtain transient absorption signals with a good signal-to-noise ratio without damaging the samples, the excitation energy of the pump light was carefully adjusted to 0.5 mW. All the experimental operations were performed at room temperature, and the same experimental conditions were steadfastly maintained throughout the experiments.

### 2.4. X-Ray Diffraction (XRD) Measurements

The X-ray diffraction (XRD) measurements were performed using the Rigaku Mini Flex 300 (Rigaku, Tokyo, Japan).

### 2.5. Steady-State Spectrometer

The steady-state UV-VIS absorption spectra of thin film samples were recorded using a UV-3101PC UV-VIS-NIR scanning spectrophotometer (Shimadzu, Tokyo, Japan).

## 3. Results and Discussion

### 3.1. Optical Properties of CsPbBr_3_

**Figure 1:**
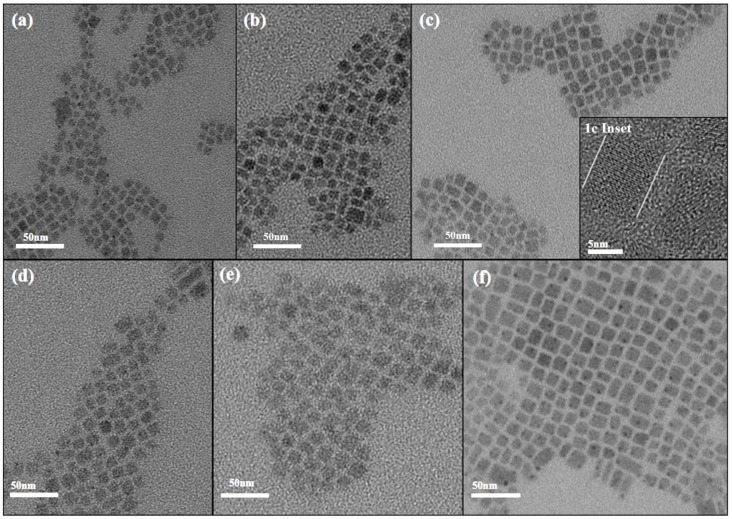
TEM images show CsPbBr_3_ nanocrystals of different sizes, including (**a**) 6.20 nm (140 °C), (**b**) 7.69 nm (150 °C), (**c**) 8.61 nm (160 °C), (**d**) 9.38 nm (170 °C), (**e**) 10.25 nm (180 °C), and (**f**) 10.68 nm (190 °C). 1c Inset: An HR TEM image of the CsPbBr_3_ at 8.61 nm.

**Figure 2:**
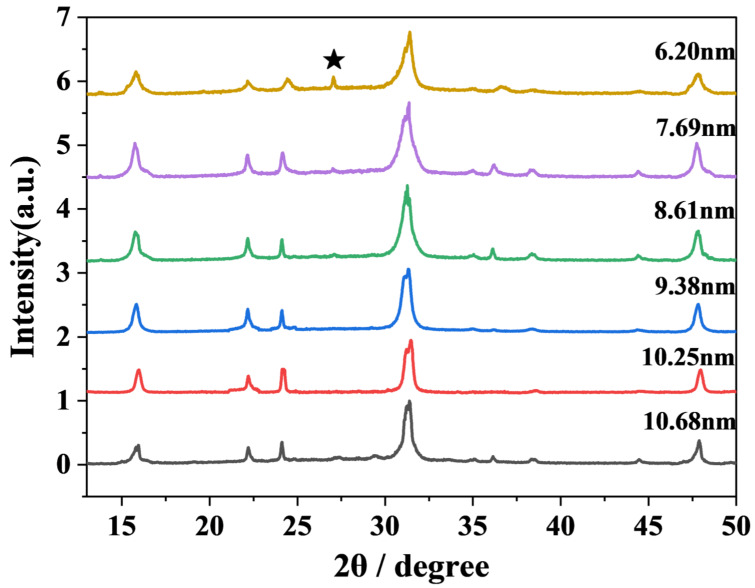
The X-ray diffraction pattern of CsPbBr_3_ at different sizes. The black pentagram pattern represents the “impurity peak”.

In order to clearly understand the morphology of CsPbBr_3_ nanocrystals, we carried out characterizations to analyze the materials. The TEM images of CsPbBr_3_ at different sizes are shown in Figure 1. As the preparation temperature increased from 140 °C to 190 °C, the size of CsPbBr_3_ nanocrystals gradually increased from 6.2 nm to 10.68 nm. The corresponding sizes were 6.20 nm (140 °C), 7.69 nm (150 °C), 8.61 nm (160 °C), 9.38 nm (170 °C), 10.25 nm (180 °C), and 10.68 nm (190 °C). The overall size of CsPbBr_3_ showed an upward trend with increasing temperature. This maintains a high degree of similarity with the experimental phenomena of Shinde et al. [32]. Three batches of CsPbBr_3_ of different sizes were synthesized at different injection temperatures. As the temperature increased, the size also became larger. The X-ray diffraction pattern of CsPbBr_3_ of different sizes is shown in Figure 2. A weak but distinguishable diffraction peak is observed at 2θ ≈ 27.2°, when the CsPbBr_3_ size is 6.20 nm. However, the peak position does not correspond to the known characteristic diffraction peaks of the CsPbBr_3_ perovskite phase. This peak may originate from precursor residues that have not been completely reacted or removed during the synthesis process. In the standard XRD (JCPDS No. 84-1181) of PbBr_2_, there is a weak diffraction peak near 27° (2θ ≈ 28.7°). If there is a slight excess of PbBr_2_ in the reaction, the unreacted residue may produce a weak peak at around 27°. It is worth noting that as the size of CsPbBr_3_ increases, the peak widths of the diffraction peaks narrow slightly. The morphological evolution of CsPbBr_3_ nanocrystals can be verified by measuring the grain size using the Scherrer equation as follows: D=kλ/βcosθ, where D is the grain size, K is a constant, λ represents the X-ray wavelength, and β and θ are the diffraction peak half-height width and diffraction angle, respectively. The size of the crystallite grains is inversely proportional to the diffraction peak half-height width [33,34]. This conclusion is consistent with what is observed in the X-ray diffraction pattern. Meanwhile, the X-ray diffraction pattern shows that different sizes of CsPbBr_3_ have the same crystal phase and do not contain any impurities. It indicates that CsPbBr_3_ perovskite materials of different sizes have been successfully prepared. The TEM images suggest that CsPbBr_3_ predominantly adopted cubic crystal structures.

**Figure 3:**
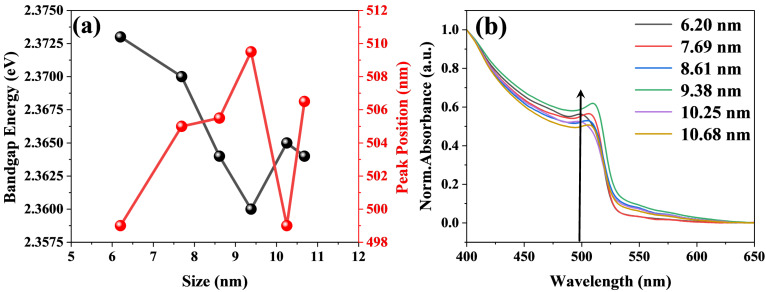
(**a**) shows the peak position and band gap energy versus crystal size curves for the assembled system of CsPbBr_3_ and TiO_2_, and (**b**) shows the steady-state absorption spectrum of assembled systems of CsPbBr_3_ of different sizes (6.20 nm, 7.69 nm, 8.61 nm, 9.38 nm, 10.25 nm, 10.68 nm) and titanium dioxide.

It is necessary to calculate the band gap using the Tauc method. As shown in Figure 3a, the band gap tends to decrease with increasing size. However, when the size of CsPbBr_3_ increases to 9.38 nm, it no longer decreases but increases instead. The steady-state absorption spectra of the assembled systems of perovskite and titanium dioxide at different sizes were obtained. As seen in Figure 3b, there is a clear absorption band in the assembled systems, which is attributed to the direct gap transition from the valence band maximum (VB) to the conduction band minimum (CB) [35,36,37]. Furthermore, the steady-state absorption peak shows a slight red shift when the size of CsPbBr_3_ increases from 6.20 nm to 9.38 nm. This phenomenon can be explained by the quantum confinement effect [38]. Given that the Bohr radius of CsPbBr_3_ is estimated to be 7 nm, the quantum confinement effect of particles larger than this size will become weak [39,40]. The reduction in the quantum confinement effect leads to a decrease in the band gap energy, which adheres to the same pattern presented in the steady-state absorption spectrum in Figure 3b [30,40,41]. It might well be the cause of the slight red shifts in the locations of the peaks. However, when the size of CsPbBr_3_ increases to around 10.25 nm, an abnormal blue shift phenomenon is observed. This is due to the built-in electric field formed by the matching band structure of the CsPbBr_3_ and TiO_2_ assembly system [42,43]. Assembling CsPbBr_3_ of different sizes with titanium dioxide may affect the built-in electric field. In addition, as shown in Figure 3a, when the size of CsPbBr_3_ increases to around 10.25 nm, the band gap shows an increasing trend. Therefore, the absorption peak exhibits a blue shift.

### 3.2. Transient Absorption Spectra of Titanium Dioxide Assembled with Perovskite of Different Sizes

**Figure 4:**
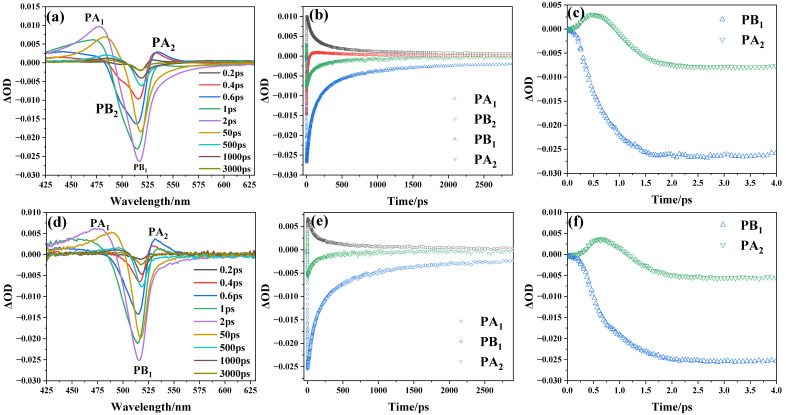
(**a**,**d**) reveals the transient absorption spectra of the 6.2 nm CsPbBr_3_ and the assembled systems of CsPbBr_3_ and titanium dioxide under different delay times; (**b**,**e**) shows the formation and decay dynamics of the characteristic signals; and (**c**,**f**) shows the dynamics of PB_1_ and PA_2_ on a short time scale.

As shown in Figure 4, in order to comprehensively understand the dynamics of interfacial charge transfer, we conducted transient absorption spectroscopy on both the CsPbBr_3_ with a size of 6.2 nm and the assembled systems of CsPbBr_3_ and titanium dioxide. Figure 4a shows the transient absorption spectra of the 6.2 nm CsPbBr_3_ under different delay times, which exhibit similar characteristics to the spectra of CsPbBr_3_ nanocrystal solutions reported by Samanta et al. [15]: a broad absorption band (labelled PA_1_) at 425–490 nm, a two-component bleach (ΔOD < 0, where ΔOD is the change in absorption) consisting of a peak at around 520 nm and a shoulder at around 500 nm (labelled PB_1_ and PB_2_ respectively), and a second absorption band (labelled PA_2_) at 530–570 nm. The accelerated recovery of PB_2_ results in spectral broadening of the PA_1_ envelope. There is striking temporal symmetry between the recovery dynamics of PB_2_ and the growth dynamics of PB_1_, which strongly suggests that hot exciton thermalization mediates the correlated bleaching phenomena. As observed in Figure 4d, compared to the transient absorption spectrum of CsPbBr_3_, the transient absorption spectrum of the assembled systems of CsPbBr_3_ and titanium dioxide shows three characteristic peaks: PA_1_, PB_1_, and PA_2_. The rapid decay of the PB_2_ signal in this system likely stems from thermal relaxation processes, in which hot carriers undergo energy dissipation to reach the lowest excitonic state, followed by interfacial electron transfer [14]. Meanwhile, Figure 4b,e illustrate that the decay of PA_2_ and the growth of PB_1_ are nearly synchronized. The same PA_2_ decay time and buildup of PB_1_ validates the hot-carrier relaxation. As shown in Figure 4c,f, the absorption signal of PA_2_ disappears after 1 ps and is eventually replaced by the PB_1_ signal. The establishment of PB_1_ is attributable to the depopulation of the ground state. The PA_1_ signal’s formation can be taken as the absorption of the lowest exciton states, and the PA_2_ signal stems from the absorption due to hot charge carriers [14].

**Figure 5:**
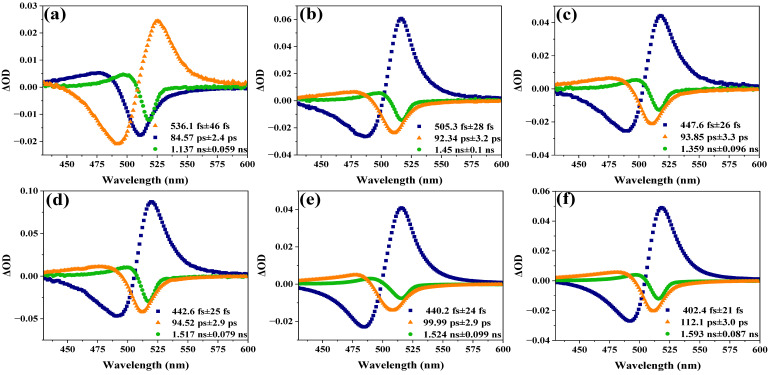
The decay-associated spectra of the assembled systems of CsPbBr_3_ and titanium dioxide with different sizes: (**a**) 6.20 nm, (**b**) 7.69 nm, (**c**) 8.61 nm, (**d**) 9.38 nm, (**e**) 10.25 nm, and (**f**) 10.68 nm.

**Figure 6:**
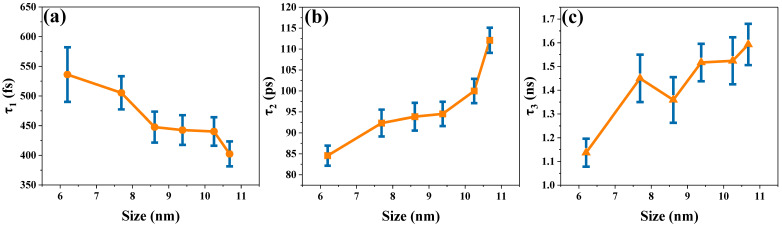
(**a**–**c**) demonstrate the time trend corresponding to the global fit of the transient absorption spectra of the assembled systems of CsPbBr_3_ and titanium dioxide with different sizes.

As shown in Figure 5, the TA spectra of the assembled systems of CsPbBr_3_ and titanium dioxide at different sizes are separated into several components by singular value decomposition (SVD) and global fitting. Taking the assembled systems of CsPbBr_3_ with a size of 6.20 nm and titanium dioxide as an example, the analysis yields three kinetic components—536.1 ± 46 fs, 84.57 ± 2.4 ps, and 1.137 ± 0.059 ns—as is evident from the decay-associated spectra (DAS) shown in Figure 5a. The time constant of 536.1 ± 46 fs represents the process of relaxation of hot carriers from a higher excited state to a lower excited state, also known as intra-band relaxation [35,44,45]. The lifetimes of electron transfer and charge recombination for CsPbBr_3_ QDs were reported to be 65 ps and 2.6 ns, respectively, by Lian et al. [46]. Therefore, the obtained time constants of 84.57 ± 2.4 ps and 1.137 ± 0.059 ns can also be attributed to the electron transfer and charge recombination processes, respectively. As shown in Table 1, the time constants of these three components are denoted as τ_1_, τ_2_, and τ_3_. It shows the time constants obtained from the transient absorption data of the assembled systems of CsPbBr_3_ and titanium dioxide with different sizes. In addition, Figure 6a–c show τ_1_, τ_2_, and τ_3_ versus the size of CsPbBr_3_.

τ_1_ is related to the cooling process of hot carriers. As Figure 6a shows, the time constant for hot-carrier cooling decreases slightly as the perovskite size increases. As the CsPbBr_3_ size changes from 6.20 nm to 10.68 nm, the corresponding time constant decreases precisely from 536.1 fs to 402.4 fs. In the assembled systems of CsPbBr_3_ and titanium dioxide, once a 400 nm pump light is incident on a sample, carriers within the CsPbBr_3_ are thermalized. Subsequently, the cooling process of hot carriers is predominantly accomplished by means of carrier–phonon coupling [47,48]. Bozyigit et al. pointed out that in PbS, the interaction between the charges and phonons can be controlled by changing the size of the nanocrystal [49]. Sum et al. simulated a nanoscale confinement approach to further delay the hot-carrier cooling of perovskite, and unlike quantum-limited conventional II-VI semiconductors, there was no manifold hole in perovskite, which would help to maintain the inherent phonon bottleneck [47]. In our experiment, as the size of perovskite increased, the lifetime of hot carriers decreased. This is because as nanocrystal size increases, the energy spacing between discrete excitonic states decreases, and the number of phonon emissions decreases, which may greatly accelerate the loss of the excess energy of hot carriers. Therefore, the time constant for the cooling of hot carriers decreases [50]. Moreover, Carlo et al. proposed that smaller sized crystals are able to keep the temperature of the carriers for a longer time, slowing down the carrier–phonon scattering (cooling) process [51], which is also similar to the conclusions reached in Figure 6a.

**Figure 7:**
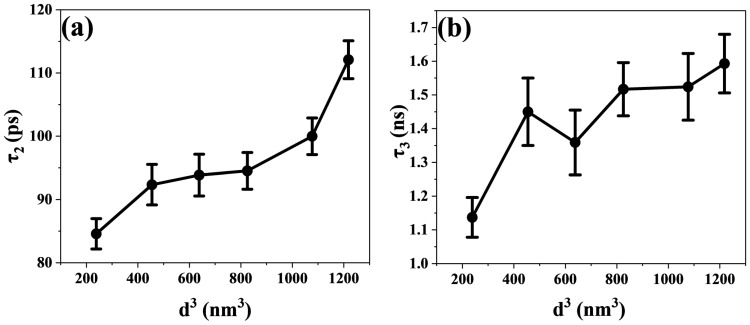
(**a**,**b**) The corresponding relationships between the time constants of transient processes and the cubic powers of the CsPbBr_3_ nanoparticle size (d^3^).

τ_2_ is related to the electron transfer process. As shown in Figure 6b, the electron transfer time constant shows an upward trend with increasing CsPbBr_3_ size. As the size of CsPbBr_3_ increases from 6.20 nm to 10.68 nm, the electron transfer time increases from 84.57 ps to 112.1 ps. The electronic band structure shows a staggered band alignment at the interface of TiO_2_ and CsPbBr_3_ [52]. In addition, the energy level difference between the CB of CsPbBr_3_ and TiO_2_ serves as the driving force for the transfer of electrons from the CB of CsPbBr_3_ to the CB of TiO_2_ [53]. Hence, the existence of the CsPbBr_3_ and TiO_2_ interface furnishes an effective transport channel for electron transfer. As shown in Figure 6b, as the perovskite size increases, τ_2_ maintains an upward trend. It has been reported that CsPbBr_3_ increases in size and that its band gap narrows slightly [54], which leads to a decrease in the energy difference between the CB of CsPbBr_3_ and that of TiO_2_. As the driving force of the electron transfer process decreases, the duration of the electron transfer from CsPbBr_3_ to TiO_2_ is prolonged. However, as shown in Figure 3a, when the size of CsPbBr_3_ nanocrystals increases to 9.38 nm, its band gap no longer decreases but increases instead. As seen in Figure 6b, the time constant of electron transfer increases more when the size of CsPbBr_3_ is larger than 9.38 nm. Here, the micelle and vesicle reaction diffusion models calculated by Tachiya are used for consideration [22,55,56]. This model describes diffusion-controlled reactions in micellar systems. We regard electron diffusion in CsPbBr_3_ nanocrystals as molecular diffusion inside micelles. As shown in Figure 7a, we plotted the relationship between the time constants of the electron transfer process and the CsPbBr_3_ nanoparticle size. It shows a nonlinear relationship (expressed as a linear function with an offset), and that a larger offset implies a slower electron transfer process. This indicates that the factors affecting the electron transfer process are not only related to the band gap.

**Figure 8:**
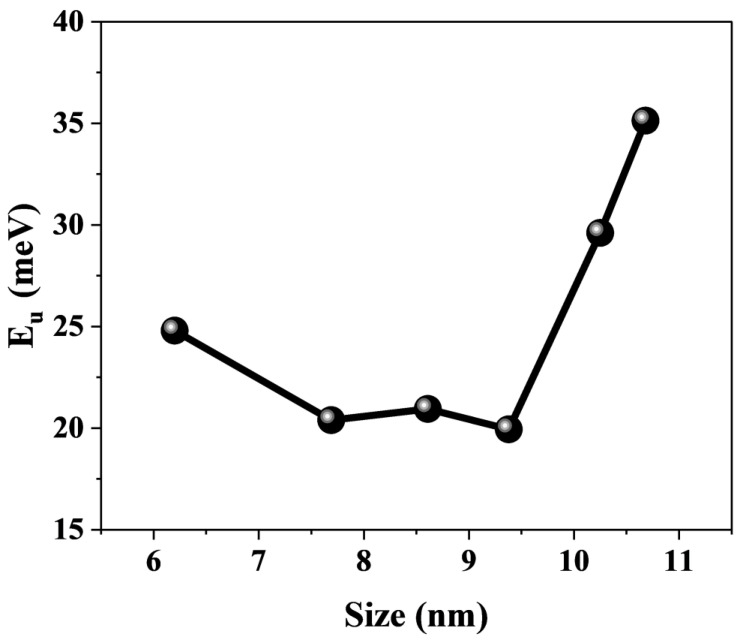
The trend of Urbach energy is observed for CsPbBr_3_ of different sizes.

**Figure 9:**
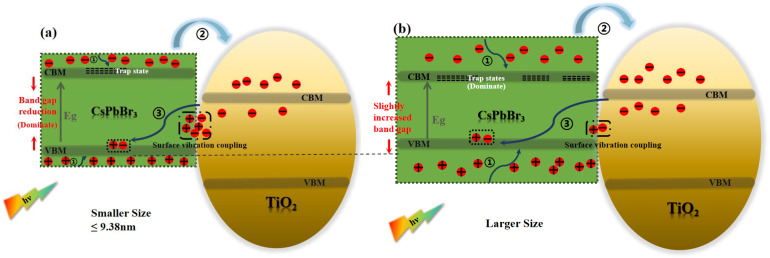
(**a**,**b**) Schematic representation of the mechanism underlying the assembled systems of CsPbBr_3_ and titanium dioxide with different sizes.

Furthermore, for the assembled systems of CsPbBr_3_ and titanium dioxide, the defect states of perovskite also affect the interfacial electron transfer process [14,53,57]. The defect states in perovskite are not located below the conduction band edge, but are located within the band edge [15,58,59,60,61]. For perovskites with the same composition, the band gap narrows as their size increases due to the quantum confinement effect. This results in lower trap state energy levels [62]. The photoexcited electrons in the perovskite are capable of being transferred to TiO_2_ by means of the trapping and detrapping mechanisms of the trap states in the conduction band. This phenomenon leads to an increase in the time constants of electron transfer. Urbach energy ($E_{U}$) is commonly used to characterize the degree of electronic disorder within semiconductor crystals, including impurities, structural defects, and electron–phonon interactions [63]. The Urbach energy was obtained by fitting the absorption spectra of the samples (Figure 3) using the following equation: $\alpha \left(E\right)=\alpha_{0}exp[\frac{E-E_{0}}{E_{U}}]$, where the photon energy E is a function of the absorption coefficient, and $E_{0}$ and $\alpha_{0}$ denote the characteristic parameters of the material. The fitting results are summarized in Figure 8. The $E_{U}$ increases as the CsPbBr_3_ size increases, and a smaller $E_{U}$ means a higher degree of crystallinity in the crystal, with fewer intrinsic and impurity defects [64]. In other words, CsPbBr_3_ with smaller sizes have fewer defect states. Additionally, smaller sizes of CsPbBr_3_ are capable of adsorbing a higher amount of organic ligands and efficiently passivating the defects of CsPbBr_3_ crystals [54]. It is also proposed that the increase in the defect state of larger sized perovskites may also be a contributing factor to the relatively long electron transfer time constant observed in these materials [33]. Therefore, at sizes of 10.25 and 10.68 nm, the defect states may become the dominant factor affecting electron transfer. This is also highly similar to the trend shown in Figure 6b. In order to gain a clear understanding of the detailed physical mechanism of electron transfer in this system and to reveal the influence of CsPbBr_3_ size, we have described a possible mechanism model, as shown in Figure 9a,b.

τ_3_ is related to the charge recombination process. The charge recombination time becomes longer as the size of the perovskite crystals increases, as shown in Figure 6c. When the CsPbBr_3_ perovskite size changes from 6.20 nm to 10.68 nm, the charge recombination time increases from 1.137 ns to 1.593 ns. The electrons will transport and be trapped in the CB of TiO_2_ following the electrons’ escape from the trap state and will continue to diffuse in TiO_2_ until they recombine with holes in the valence band of the perovskite [65]. Similarly, the Tachiya model has also been considered for analyzing the charge recombination process. In Figure 7b, the nonlinear relationship between the time constant of the charge recombination process and the size of CsPbBr_3_ nanoparticles is also shown. This proves that there is more than one factor influencing the charge recombination process. The diffusion length of carriers and the localized state of the interface collectively affect the interfacial charge recombination process [14,35,66]. It has been found that the time of charge recombination is related to the length of electron diffusion. As the electron diffusion length increases, the charge recombination time constant also increases [22]. In addition, Du’s work found that the diffusion length of electrons is positively correlated with the particle size of nanoparticles [56]. In our experiment, we observed that as the size of CsPbBr_3_ increased, the time constant of charge recombination showed an upward trend. Therefore, the prolonged diffusion length of carriers was among the reasons leading to the prolonged recombination time. As shown in Figure 9a,b, we have also described a possible mechanism model. Furthermore, charge recombination occurs in the localized states at the interface [14]. As the perovskite size increases, a larger proportion of the electron and hole charge density will be coupled to the “interior” of the NC (with less coupling to surface vibrational modes). This should lead to a decrease in symmetry breaking and an increase in charge recombination time constants [67]. This is also consistent with the data trend shown in Figure 6c. The increase in charge recombination times indicates that electrons and holes are not easily recombined, which may also indicate that the perovskite layer with the longer charge lifetime is a higher-quality film [68]. The above research results indicate that large-sized CsPbBr_3_ perovskite is more conducive to improving the photoelectric conversion efficiency of all inorganic perovskite solar cells.

## 4. Conclusions

In summary, we manipulated the size of the CsPbBr_3_ by altering the synthesis temperature. Moreover, femtosecond ultrafast pump probe technology was used to detect the transient dynamics of the assembled systems of CsPbBr_3_ and titanium dioxide with different sizes. Upon carrying out singular value decomposition (SVD) and global fitting on the femtosecond transient absorption spectrum data, three dynamic components were identified: the relaxation of hot carriers, interfacial electron transfer, and charge recombination. According to the experimental data, as the size of CsPbBr_3_ increases, the phonon bottleneck effect decreases, and then, the time constant of the hot-carrier cooling process decreases. In addition, as the size of the perovskite increases, the defect states of CsPbBr_3_ increase and the energy difference between CsPbBr_3_ CB and TiO_2_ CB decreases, resulting in an increasing trend in the interfacial electron transfer time. The increase in charge recombination time is due to the increase in perovskite size, which leads to an increase in carrier diffusion length and changes in interfacial localized states. The above results suggest that increasing the size of perovskite improves its photoelectric conversion efficiency and helps to provide physical-theoretical guidance for the design of high-efficiency quantum dot solar cells.

## Figures and Tables

**Table 1:** Time constants obtained from the global fitting results of the assembled systems of CsPbBr_3_ and titanium dioxide with different sizes.

Size (nm)	τ_1_ (fs)	τ_2_ (ps)	τ_3_ (ns)
6.20	536.1 ± 46	84.57 ± 2.4	1.137 ± 0.059
7.69	505.3 ± 28	92.34 ± 3.2	1.450 ± 0.100
8.61	447.6 ± 26	93.85 ± 3.3	1.359 ± 0.096
9.38	442.6 ± 25	94.52 ± 2.9	1.517 ± 0.079
10.25	440.2 ± 24	99.99 ± 2.9	1.524 ± 0.099
10.68	402.4 ± 21	112.1 ± 3.0	1.593 ± 0.087

## Data Availability

The data that support the findings of this study are available from the corresponding authors upon reasonable request.
